# Modulation of proinflammatory activity by the engineered cationic antimicrobial peptide WLBU-2

**DOI:** 10.12688/f1000research.2-36.v1

**Published:** 2013-02-08

**Authors:** Shruti M Paranjape, Thomas W Lauer, Ronald C Montelaro, Timothy A Mietzner, Neeraj Vij

**Affiliations:** 1 Eudowood Division of Pediatric Respiratory Sciences, Department of Pediatrics, John Hopkins University, Baltimore, MD, 21287, USA; 2 Department of Microbiology and Molecular Genetics, University of Pittsburgh, Pittsburgh, PA, 15261, USA; 3 Center for Vaccine Research, University of Pittsburgh, Pittsburgh, PA, 15261, USA; 4 Lake Erie College of Osteopathic Medicine, Seton Hill University, Greensburg, PA, 15601, USA

## Abstract

**Background:** Host-derived (LL-37) and synthetic (WLBU-2) cationic antimicrobial peptides (CAPs) are known for their membrane-active bactericidal properties. LL-37 is an important mediator for immunomodulation, while the mechanism of action of WLBU-2 remains unclear.

**Objective:** To determine if WLBU-2 induces an early proinflammatory response that facilitates bacterial clearance in cystic fibrosis (CF).

**Methods:** C57BL6 mice were given intranasal or intraperitoneal 1×10 ^6^ cfu/mL *Pseudomonas aeruginosa* (PA) and observed for 2h, followed by instillation of LL-37 or WLBU-2 (2-4mg/kg) with subsequent tissue collection at 24h for determination of bacterial colony counts and quantitative RT-PCR measurement of cytokine transcripts. CF airway epithelial cells (IB3-1, ΔF508/W1282X) were cultured in appropriate media with supplements. WLBU-2 (25μM) was added to the media with RT-PCR measurement of TNF-α and IL-1β transcripts after 20, 30, and 60min. Flow cytometry was used to determine if WLBU-2 assists in cellular uptake of Alexa 488-labeled LPS.

**Results:** In murine lung exposed to intranasal or intraperitoneal WLBU-2, there was a reduction in the number of surviving PA colonies compared to controls. Murine lung exposed to intraperitoneal WLBU-2 showed fewer PA colonies compared to LL-37. After 24h WLBU-2 exposure, PA-induced IL-1β transcripts from lungs showed a twofold decrease (p<0.05), while TNF-α levels were unchanged. LL-37 did not significantly change transcript levels. In IB3-1 cells, WLBU-2 exposure resulted in increased TNF-α and IL-1β transcripts that decreased by 60min. WLBU-2 treatment of IB3-1 cells displayed increased LPS uptake, suggesting a potential role for CAPs in inducing protective proinflammatory responses. Taken together, the cytokine response, LPS uptake, and established antimicrobial activity of WLBU-2 demonstrate its ability to modulate proinflammatory signaling as a protective mechanism to clear infection.

**Conclusions:** The immunomodulatory properties of WLBU-2 reveal a potential mechanism of its broad-spectrum antibacterial activity and warrant further preclinical evaluation to study bacterial clearance and rescue of chronic inflammation.

## Introduction

Cationic antimicrobial peptides (CAPs) are one effector of the innate immune response, the “first line of defense” against a pathogenic insult. They are ancient, structurally diverse elements of the immune responses of all living species. These molecules typically have broad-spectrum antimicrobial activity with conserved recognition patterns to molecules such as lipopolysaccharide (LPS) and lipoteichoic acid. They are rapidly induced on the order of minutes to hours. Their amphipathic structures facilitate their antimicrobial killing activity at the level of the bacterial membrane. It has become increasingly apparent that CAPs also play key roles in inflammatory responses and in orchestrating the mechanisms of innate immunity ^1^.

LL-37, or human cathelicidin, is a CAP that has been localized to airway epithelium. In its mature form, it is an α-helical peptide made up of 37 residues that has been shown to possess broad spectrum antibacterial activity as well as other host defense functions such as chemotaxis, LPS neutralization, angiogenesis, and wound healing. First cloned from a bone marrow library, the expression of this peptide has been detected in many epithelial tissues, including the testes, epidermis, and the gastrointestinal and respiratory tracts ^2^. The antibacterial and immunomodulatory roles of the cathelicidins are currently under intense investigation and appropriate models of infection are required for understanding their contribution to host defense.

LL-37 is also an important modulator of the human immune response. This host-derived CAP is an antiseptic agent with the ability to inhibit macrophage stimulation by bacterial components such as LPS, lipoteichoic acid, and noncapped lipoarabinomannan ^3^. Using gene expression profiling to identify potential LL-37-modulated macrophage functions, LL-37 directly upregulated 29 genes and downregulated another 20 genes. Among the genes predicted to be upregulated by LL-37 were those encoding chemokines and chemokine receptors. Consistent with this, LL-37 upregulated the expression of chemokines in macrophages and the mouse lung (monocyte chemoattractant protein 1), human A549 epithelial cells (IL-8), and whole human blood (monocyte chemoattractant protein-1 and IL-8), without stimulating the proinflammatory cytokine TNF-α. LL-37 also upregulated the chemokine receptors CXCR-4, CCR2, and IL-8RB. It appears that LL-37 contributes to the immune response by limiting the damage caused by bacterial products and recruiting immune cells to the site of infection ^4^.

WLBU-2, by comparison, is a completely synthetic α-helical, engineered CAP (eCAP) made up of a repeating sequence of Arg, Lys, and Trp residues ^5– 7^. Previous work has demonstrated this compound’s broad spectrum antibacterial activity against bacterial pathogens in both *in vivo* and *in vitro* systems ^5, 6, 8^. Among the many antimicrobial peptides currently described in the literature, eCAPs are most chemically and structurally homologous to the magainins ^9^ and LL-37. They are peptides of approximately 30 residues that, when modeled as an α-helix, demonstrate amphipathic character with defined cationic and hydrophobic faces. The selectivity of the eCAPs for bacterial membranes, like other host-derived CAPs, presumably results from their affinity for negatively charged lipids found on the bacterial surface. The high-energy potential of the bacterial membrane facilitates self-promoted CAP uptake, thus compromising the integrity of the bacterial cell by disrupting the lipid bilayer ^10^ and suggests that these α-helical peptides ^11^ may be suitable agents for treating bacterial airway infections.

Despite extensive characterization of the antimicrobial activity of the eCAPs, little is known about their immunomodulatory properties ^5, 6^. The purpose of this study was to determine if WLBU-2 modulates an early proinflammatory response to facilitate *Pseudomonas aeruginosa* (PA) clearance using both *in vivo* and *in vitro* models. The *in vivo* model partially replicates some of the phenotypic lung disease of CF in mice that resemble wild-type animals in size and survival. Development of a murine model of lung inflammation is highly desirable to accelerate pre-clinical testing of novel anti-inflammatory therapeutics. These studies demonstrate the immunomodulatory properties of an engineered, synthetic compound not only for the purpose of its potential development as a novel antibacterial agent, but also for its contribution to study the role of α-helical peptides in the processes of host defense.

## Methods

### Cell culture and treatments

For the *in vitro* studies, CF airway epithelial cells (IB3-1, ΔF508/W1282X, American Type Culture Collection (ATCC), Manassas, VA) were cultured and maintained in LHC-8 medium containing 10% FBS, 100 units/mL penicillin, and 100 μg/mL streptomycin. WLBU-2 (25μM) was added to the media with qRT-PCR measurement of TNF-α and IL-1β transcripts after 20, 30, and 60min. IB3-1 cells were used for flow cytometry ^12^ and measurement of proinflammatory cytokine activity. Transfected cells containing a wild-type or NFκB mutant-IL-8 promoter gene ^13^ were treated with WLBU-2 followed by measurement of IL-8 reporter activity.

### IL-8 promoter activity studies

These studies used CF IB3-1 (ΔF508/W1282X) cells, transiently transfected with α 5' firefly luciferase gene (Clontech Laboratories, Inc, Mountain View, CA) flanking a wild type- or NFκB mutant-IL-8 promoter, using Lipofectamine 2000 (Invitrogen, Carlsbad, CA) for 24h as previously described ^13, 14^. The CAP LL-37 (25μM) or the eCAP WLBU-2 (25μM) was added to the media for 30min-4h. The Dual-Luciferase ^®^ Reporter (DLRTM) Assay System (Clontech Laboratories, Inc, Mountain View, CA) was used to measure IL-8 reporter activity in IB3-1 cells. *Renilla* luciferase (Clontech Laboratories, Inc, Mountain View, CA) was used as an internal control to normalize changes in IL-8 promoter-driven firefly luciferase activity across the samples.

### Flow cytometry

IB3-1 cells were incubated overnight at 37°C with LPS-Alexa Fluor 488 (Invitrogen, Carlsbad, CA) and Lipofectamine 2000. PBS or WLBU-2 (25µM) was added to the flask for 24h. After the addition of Cell Dissociation Buffer (Invitrogen, Carlsbad, CA), cells were collected, centrifuged, and rinsed with PBS. Finally, cells were treated with FIX & PERM (Invitrogen, Carlsbad, CA) per the manufacturer’s directions. Flow cytometry was done on a BD FACS can flow cytometer and data were analyzed using CellQuest.

### Murine studies

This protocol was approved by the institutional Animal Care and Use Committee (ACUC). Animals used in this study were followed for 1–5 days (4 animals per cage) in approved satellite housing to allow close observation from the beginning to end of each experiment. Feeding practices, light cycle, and temperature and humidity, and cage and room cleaning procedures were identical to those of this institution’s central animal facility in accordance with ACUC recommendations. Animals were studied in four groups of four animals per experiment. The groups consisted of a) control C57/BL6 wild-type mice; b) C57/BL6 wild-type mice receiving bacteria (positive control); c) the eCAP WLBU-2 or the CAP LL-37 (test agents); or d) the combination of bacteria and WLBU-2 or LL-37. Animals in the experimental groups received the test agents, while control animals received sterile phosphate-buffered saline via the trachea using the posterior pharyngeal approach. These studies were carried out in the presence or absence of a proinflammatory stimulus (bacterial exposure).

Mice were anesthetized using a vaporizer set to deliver a mixture of 3% isoflurane and oxygen in preparation for the instillation of control or test agents into the respiratory tree. Complete anesthesia was determined by visual confirmation of a slowed respiration rate and the animal’s response to other clinical tests such as leg withdrawal and tail pinch. Animals were weighed prior to the procedure. The method is based on a published study ^15^. Once anesthetized, the tongue was gently extruded using padded forceps and the control and/or test agents (volume 50µL) were pipetted into the posterior section of the oral cavity. The positive control was 1) *P. aeruginosa* (ATCC type strain, 1×10 ^6^ cfu/mL); the negative control was PBS. The test agents were the host-derived CAP LL-37 or the engineered CAP WLBU-2 (dose 1mg/kg). Following aspiration of the test agent, 100% O _2_ was given until the animal awakened. Animals were then placed into a 37°C chamber until completely recovered.

Intraperitoneal PA infection in age (8 weeks) and sex-matched C57BL/6 mice was established ^16^ followed by WLBU-2 (4mg/kg), LL-37 (4mg/kg), or PBS treatment ^5^, followed by collection at 24h of bronchoalveolar lavage (BAL) samples and lung tissue for bacterial culture on trypticase soy agar plates, quantitative RT-PCR, ELISA, and microscopy ^12^. Mice were anesthetized with 3% isoflurane before and during treatment. After 24h, mice were sacrificed for sample collection.

At the endpoint of the experiments mice were anesthetized using a vaporizer set to deliver a mixture of 3% isoflurane and oxygen and weighed. Once completely anesthetized, animals were euthanized by cervical dislocation followed by collection of BAL samples from within an exposed thorax using sterile phosphate-buffered saline (PBS) through a cannula inserted in the trachea. BAL samples were used for protein analysis as well as leukocyte differentiation. Lungs were excised and then stored in buffers or preservatives for protein analysis or histologic examination.

Mouse lungs were homogenized in TRIzol (Invitrogen, Carlsbad, CA) and processed for isolation of total RNA according to the manufacturer's instructions. The Super Script III First-Strand Synthesis System (Invitrogen, Carlsbad, CA) was used to catalyze the reverse transcription reaction from 1µg of total RNA. Quantitative RT-PCR (qRT-PCR) was performed using TaqMan Gene Expression Master Mix and TaqMan Gene Expression Assays (Applied Biosystems, Foster City, CA) for GAPDH (control reagent), IL-1β, and TNF-α (4352932, Mm01336189_m1, Mm99999068_m1, respectively). The qRT-PCR reactions were amplified using an ABI PRISM 7700 Sequence Detection System. Relative gene expression was determined using ∆∆Ct calculations.

### ELISA and immunohistology

ELISA kits for IL-1β and IL-6 were obtained from R&D Systems (Minneapolis, MN). The manufacturer’s protocol was followed for the detection of each cytokine in 10µL of BAL fluid as well as in standardized samples provided in the kit. SoftMax Pro (Molecular Devices, Sunnyvale, CA) was used to read the 96-well plates in a Molecular Devices VersaMax microplate reader. A plot of the standardized samples was used to calculate cytokine expression in the BAL samples. For histologic examination, lungs were fixed overnight in 4% paraformaldehyde at 4°C and subsequently embedded in paraffin. Tissue sections (5µm thickness) were deparaffinized and rehydrated with xylenes and a series of decreasing ethanol concentrations, respectively. Hematoxylin and eosin were used to stain the sections prior to microscopic examination.

## Results

### CAPs modulate proinflammatory cytokine activity *in vitro* in the absence of bacteria

**Figure 1.**
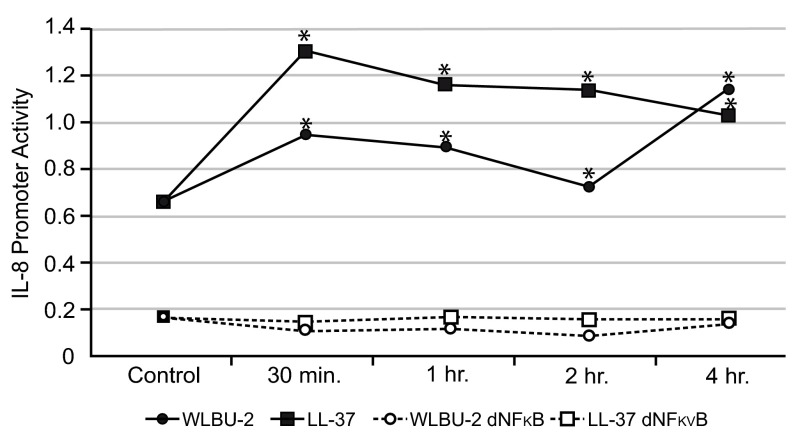
WLBU-2 increases IL-8 promoter activity in CF IB3-1 cells. CF IB3-1 (ΔF508/W1282X) cells transiently transfected with a 5' firefly luciferase gene flanking a 200bp wild type- *(solid lines)* or NFκB mutant-IL-8 promoter *(dashed lines)* were treated with 25µM WLBU-2 *(solid circles)* or 25µM LL-37 *(solid squares)* followed by measurement of IL-8 promoter activity at time points ranging from 30min-4h by Dual-Luciferase Reporter assay. There was a significant increase in IL-8 promoter activity in cells exposed to either peptide compared to control ( *asterisks, p*<0.005). In cells lacking the NFκB site *(dashed lines)*, there was little IL-8 promoter activity, suggesting that both WLBU-2 *(open circles)* and LL-37 *(open squares)* may influence IL-8 secretion through the NFκB pathway.

**Figure 2.**
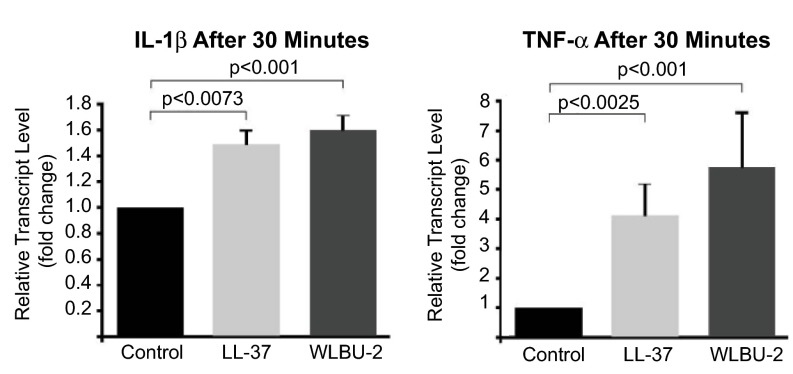
CAPs modulate proinflammatory cytokine activity *in vitro* in the absence of bacteria. CF IB3-1 cells were cultured in LHC-8 media with supplements, followed by treatment with PBS *(black bars)*, LL-37 (25µM, *light grey bars*) or WLBU-2 (25µM, *dark grey bars*) and measurement of the levels of the proinflammatory cytokines IL-1β and TNF-α by quantitative RT-PCR at time points varying from 0–60min. Data represent fold-change in relative transcript levels of proinflammatory cytokines obtained 30min after peptide exposure; both IL-1β and TNF-α showed significant increases in relative transcript levels that decreased by 60min (see raw data file). CAPs, as effector molecules of the innate immune response, may exert an initial protective effect at the level of the epithelial surface against a potential pathogenic or inflammatory challenge.

IB3-1 cells were used to examine proinflammatory cytokine effects after WLBU-2 exposure. IL-8 promoter activity was significantly increased compared to control in both WLBU-2-exposed IB3-1 cells after 4h (mean±SD 1.14±0.0004 *vs.* 0.67±0.0002, *p*<0.005, Figure 1) and LL-37-exposed cells (mean±SD 1.04±0.0006 *vs.* 0.67±0.0002, Figure 1). WLBU-2-exposed cells showed significantly less IL-8 promoter activity compared to LL-37-exposed cells ( *p*<0.005). IB3-1 cells with an NFκB mutant IL-8 promoter exposed to either peptide showed minimal reporter activity at 4h ( Figure 1), suggesting that both WLBU-2 and LL-37 influence IL-8 secretion through NFκB. Exposure to either WLBU-2 (25μM) or LL-37 (25μM) for 30min ( Figure 2) resulted in increased transcript levels of IL-1β (mean±SD fold-change WLBU-2 1.59±0.12; LL-37 1.47±0.12, *p*<0.005) and TNF-α (mean±SD fold-change WLBU-2 5.74±1.83; LL-37 4.11±1.07, *p*<0.005) compared to control and decreased by 60min toward baseline (see raw data file).

**Figure 3.**
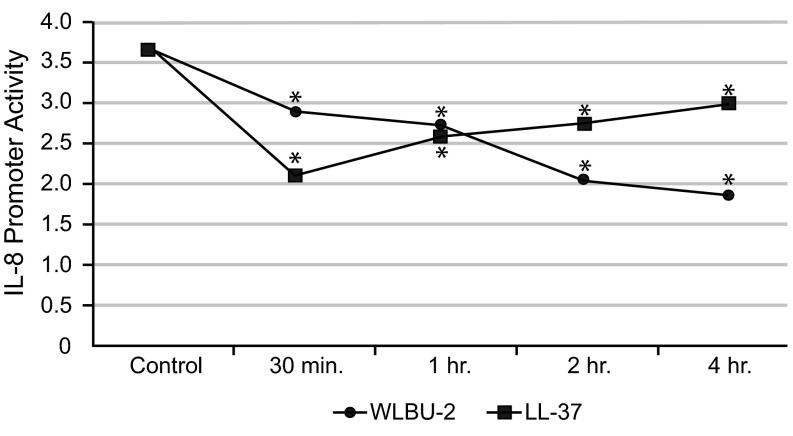
eCAPs show decreased *in vitro* IL-8 promoter activity in LPS-stimulated cells. CF IB3-1 (ΔF508/W1282X) cells were transiently transfected with a 5' firefly luciferase gene flanking a 200bp wild type-IL-8 promoter. After application of *Pseudomonas aeruginosa* LPS, WLBU-2 (25µM, *circles*) or LL-37 (25µM, *squares*) was added to the plate followed by measurement of IL-8 promoter activity at time points ranging from 30min-4h by Dual-Luciferase Reporter assay. Promoter activity is plotted for both peptides with an LPS-stimulated control. Both peptides showed a significant decrease in LPS-induced IL-8 promoter activity ( *asterisks, p*<0.005); the eCAP WLBU-2 showed a sustained decrease over 4h while LL-37 showed an increasing trend, suggesting that eCAPs may be beneficial as immunomodulators as well as antibacterial agents.

Using equimolar (25μM) peptide concentrations in LPS-stimulated cells, WLBU-2 showed less LPS-induced IL-8 reporter activity compared to LL-37 after 4h (mean±SD 1.82±0.0034 *vs.* 2.93±0.0009, *p*<0.005, Figure 3). By flow cytometry, WLBU-2-exposed cells showed 91% uptake of fluorescently labeled LPS after 24h compared to 75% of control cells. These results suggest that WLBU-2 induces a protective inflammatory response through interaction with LPS (T. Mietzner, unpublished observation) and exhibits an initial protective effect against epithelial inflammatory challenges.

Proinflammatory cytokine measurements in IB3-1 cells following cationic antimicrobial peptide exposure IB31 cells were exposed to 25μM LL37 or WLBU2 for various amounts of time, followed by quantitative RT-PCR measurement of IL1β & TNFα transcripts. The spreadsheet includes raw data and delta-delta Ct calculations for Real-time PCR; strikethrough values were not included in the analyses. GADPH was used as a control reagent.

### Modulation of *P. aeruginosa*-induced proinflammatory cytokine responses by WLBU-2

**Figure 4.**
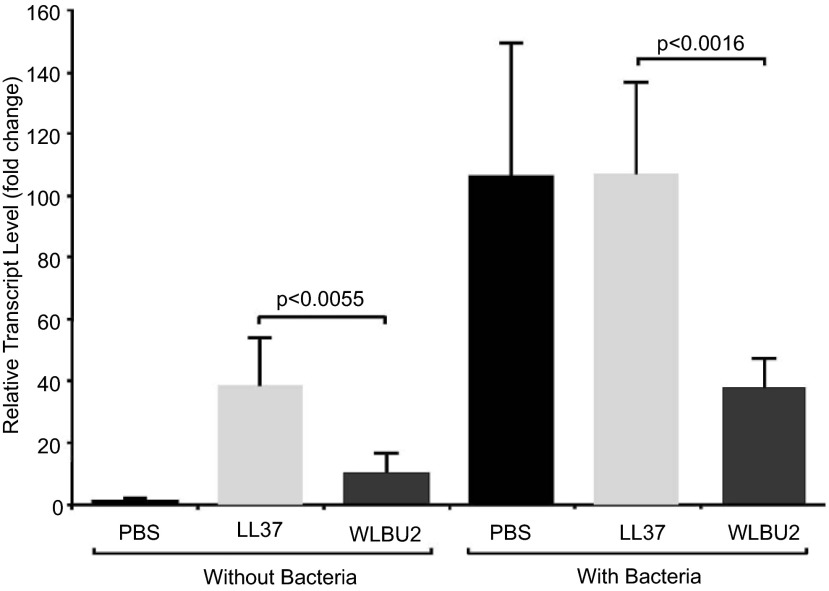
Intraperitoneal WLBU-2 suppresses IL-1β in lungs systemically exposed to *P. aeruginosa* (PA). Wild-type C57BL/6 animals (n=5/group) received intraperitoneal injections of PBS or PA (1×10 ^6^ cfu/mL), followed by intraperitoneal injections after 2h of PBS *(black bars)*, LL-37 (4mg/kg, *light grey bars*), or WLBU-2 (4mg/kg, *dark grey bars*), with subsequent measurement by quantitative RT-PCR of IL-1β transcripts ( *y* axis, fold change in relative transcript level) from lung tissue harvested 24h post exposure. Data represent measurements performed in triplicate. In groups not receiving bacteria, LL-37 and WLBU-2-exposed animals showed an increase in IL-1β transcripts compared to PBS controls. In groups receiving PA, WLBU-2-exposed animals showed significant suppression of IL-1β ( *p*<0.005) compared to LL-37. These data suggest that the eCAP WLBU-2 may modulate proinflammatory cytokine release in the setting of acute infection.

WLBU-2 treatment of animals (n=5/group) with intraperitoneal PA infection showed more IL-1β suppression compared to LL-37 (mean±SD fold-change 37.6±9.7 *vs.* 105.9±30.9, *p*<0.005, Figure 4). There was no effect on TNF-α (data not shown). Consistent with prior efficacy studies comparing the bactericidal activities of WLBU-2 and LL-37 ^5, 6^, bacterial cultures of lung homogenates from WLBU-2-treated animals showed no growth compared to those given LL-37 after 24h (0 cfu/mL *vs*. 5800 cfu/mL).

IL-8 Reporter Assay in LPS-stimulated and unstimulated cystic fibrosis airway cells treated with WLBU-2 or LL-37 CF IB3-1 cells (unstimulated and LPS-stimulated) were transfected with a 5' firefly luciferase gene flanking a 200bp wild type- or dNFκB IL-8 promoter and treated with 25µM WLBU-2 or LL-37 followed by measurement of IL-8 promoter activity at time points ranging from 30min-4h by Dual-Luciferase Reporter assay. PBS was used as a control in LPS-stimulated and unstimulated cells. Data represent means/standard deviations of the count readings and calculations of fold-change relative to the PBS-treated controls.

## Discussion

Previous CAP studies have demonstrated a dose-dependent decrease in LPS-activated neutrophilic proinflammatory cytokine release by LL-37 ^17^. Neutrophils stimulated by heat-inactivated bacteria showed a decrease in TNF-α after LL-37 exposure, whereas those from cathelicidin-deficient mice showed less antimicrobial activity and increased proinflammatory cytokine release, suggesting that endogenous cathelicidin modulates the neutrophilic innate immune response. Since Toll-like receptor signaling effects in macrophages differ depending on exogenous or endogenous peptide origin and cellular activation state, exogenous cathelicidin application resulted in blunted activation of p38 and ERK MAPKs and decreased TNF-α release in macrophages exposed to LPS and reversed diminished MAPK activation associated with LPS tolerance. Endogenous cathelicidin release from macrophages in cathelicidin-deficient animals neither inhibited LPS MAPK and cytokine activation nor rendered animals more susceptible to lethal LPS challenges ^18^. Other studies demonstrated that LL-37 and LPS interactions alter endotoxin aggregation, which may explain the observed inhibition of proinflammatory activity ^19– 21^.

eCAPs represent a novel class of effective antimicrobial peptides ^6, 22– 25^ that demonstrate broad-spectrum activity against highly resistant bacterial strains ^22^. The antibacterial efficacy of WLBU-2 using murine models of intraperitoneal infection and bacteremia has been described ^5, 6^. Intravenous WLBU-2 effectively treated systemic PA infection and was protective when administered 1h prior to establishing bacteremia. Animals treated with subtherapeutic doses of WLBU-2 showed lower IL-1β and TNF-α levels after 3–5h compared to animals exposed to heat killed bacteria (T. Mietzner, unpublished observation). Despite demonstration of *in vivo* efficacy, further studies are needed to define the immunomodulatory properties of WLBU-2 in the setting of respiratory infection.

This paper advances previous work on WLBU-2 by defining its antibacterial and immunomodulatory activities *in vivo* and *in vitro*. The *in vitro* studies demonstrate constitutive changes in proinflammatory signaling in the absence of bacteria. The increased LPS uptake in CF epithelial cells indicates the possibility of an LPS interaction as an immunomodulatory function but may have been limited by cellular toxicity resulting from prolonged peptide exposure ^8^. Because peptide inactivation through interaction with LPS may affect cellular uptake ^26^, the optimized hydrophobicity of WLBU-2 could contribute to more effective LPS neutralization ^27^.

The cytokine response, LPS uptake, and established antimicrobial activity of WLBU-2 demonstrate modulation of proinflammatory signaling as a protective mechanism to clear infection. Because *in vivo* studies of WLBU-2 have demonstrated effective bacterial killing activity and proinflammatory cytokine suppression, further work examining CAPs in innate immune responses will assist in defining the roles of CAPs in host defense and of eCAPs in the development of novel antibacterial and immunomodulatory therapies.
