# Supplementary material for: Modulation of proinflammatory activity by the engineered cationic antimicrobial peptide WLBU-2
Source: F1000Res. 2013 Feb 8;2:36. [Version 1] doi: 10.12688/f1000research.2-36.v1 (PMC3894802; doi:10.12688/f1000research.2-36.v1)
Supplement: Peer review history

# Peer review: Referee response for version 1

**Published:** 2013-10-25

**Authors:** Paul Savage

**Affiliations:** 1 Department of Chemistry and Biochemistry, Brigham Young University, Provo, UT, USA

This manuscript makes a correlation between the antibacterial and immunomodulatory effects of LL-37 and the synthetic antimicrobial peptide WLBU-2. The authors find that WLBU-2 stimulates cytokine (IL-8) production, which is likely to impact bacterial clearance. This observation implies that the WLBU-2 is interacting comparably with receptors that respond to the presence of LL-37. Overall, the manuscript is well written, the data are well presented, and though the body of work is modest, it should be of interest to a relatively large group of researchers.

I have read this submission. I believe that I have an appropriate level of expertise to confirm that it is of an acceptable scientific standard.

# Peer review: Referee response for version 1

**Published:** 2013-10-07

**Authors:** Anastasia Nijnik

**Affiliations:** 1 Department of Physiology and the Complex Traits Group, McGill University, Montréal, Canada

The study presents novel and convincing data for immunomodulatory activity of synthetic cationic peptide WLBU-2. Specifically, the authors demonstrate that WLBU-2 induces IL-8 promoter activity and stimulates TNFa and IL1b gene expression in the IB3-1 cystic fibrosis (CF) airway epithelial cells. Several questions remain to be addressed. In particular, given that TNFa and IL1b production is regulated at post-transcriptional level, it will be important to address whether WLBU-2 stimulates the production and secretion of TNFa, IL1b and/or IL8 proteins. Also, given the relatively high concentration of peptide used in the *in vitro* studies, it is important to address whether the induction of inflammatory cytokines could be linked to non-specific cytotoxic effects of the peptide on the epithelial cells.

The IB3-1 cystic fibrosis (CF) airway epithelial cells were chosen for the study because immunomodulatory peptides are being explored as a potential therapeutic against bacterial colonization and inflammatory tissue damage in CF. At present it is unclear whether there are any differences between wild type and IB3-1 epithelial cells in their responses to WLBU-2. Possible immunomodulatory activities of WLBU-2 on other cell types (e.g. leukocytes) also remain to be addressed. These are possible interesting directions for future studies.

The paper is clearly written. Given the many previous studies exploring anti-endotoxic properties of cathelicidins, and activities of cationic peptides in CF models, some sections of the paper could benefit from additional citations.

I have read this submission. I believe that I have an appropriate level of expertise to confirm that it is of an acceptable scientific standard.
